# Cost-effectiveness of testing biofluid biomarkers to diagnose Alzheimer’s disease: a systematic review

**DOI:** 10.1007/s10198-025-01877-1

**Published:** 2025-12-12

**Authors:** Hanzi Jiang, Sergio Escamilla, Molly Beestrum, Federico Salas-Lucia

**Affiliations:** 1 Institute of Public Health and Medicine, Northwestern University Feinberg School of Medicine, 633 N. St Clair; 20th Floor, Chicago, IL 60611 USA; 2 Department of Medicine, Section of Adult and Pediatric Endocrinology and Metabolism, University of Chicago, Chicago, IL USA; 3 Neuroscience Institute, University of Chicago, Chicago, USA

**Keywords:** Cerebrospinal fluid biomarkers, Blood-based biomarkers, Economic evaluation, Model-based simulation

## Abstract

**Background:**

Alzheimer’s disease (AD) affects tens of millions of individuals and their families in the world. As AD progresses, the effectiveness of treatment declines, making a timely diagnosis crucial. One promising approach for timely diagnosis is testing biofluid biomarkers in patients’ blood and cerebrospinal fluid (CSF). However, a comprehensive evaluation of whether these tests are cost-effective is missing. Here, we conducted a systematic review to assess the cost-effectiveness of testing biofluid biomarkers to diagnose AD.

**Method:**

We searched PubMed, Embase, Cochrane Library, and Web of Science for studies published until October 2024. Studies were included if published in English, conducted an economic evaluation of CSF or blood biomarker testing to diagnose AD, and provided economic outcomes. We assessed the quality of studies using the CHEERS 2022 criteria.

**Results:**

Nine studies were included: six evaluated CSF biomarker tests and three on blood biomarker tests. All studies used model-based simulations (decision trees or Markov models) rather than trial-based evaluations. CSF biomarker tests were mostly cost-effective compared to neurocognitive assessments or neuroimaging, while blood biomarker tests showed mixed results. Key cost-effectiveness contributors included AD prevalence, diagnostic accuracy, and treatment effectiveness. Studies met ~ 80% of the CHEERS criteria, with missing information on patient engagement.

**Conclusion:**

Our review supports that biofluid biomarker testing could be cost-effective to diagnose AD. Given the lack of trial-based economic evaluations, model-based studies are a valuable starting point. Future evaluations should incorporate patient-centered outcomes and consider the emotional value and other socio-economic factors that affect patients and families suffering from AD.

**Supplementary Information:**

The online version contains supplementary material available at 10.1007/s10198-025-01877-1.

## Introduction

 Alzheimer’s disease (AD) is a devastating neurodegenerative disorder, affecting the lives of tens of millions of individuals and their families worldwide [1]. In Europe alone, it was estimated that 5% of the population is living with AD, a figure that is continuing to rise with the aging population [2, 3]. In its early stages, the disease often manifests as sporadic loss of memory and cognitive decline [4]. As AD progresses, the cognitive impairments become more severe, including a total loss of the patient’s behavioral abilities to function independently, drastically reducing their quality of life [5]. For patients with AD, timely and accurate diagnosis of AD is paramount as it facilitates appropriate treatments that may slow the progression of the disease and improve patient outcomes [6]. Delays in diagnosis could hold up treatment and limit treatment effectiveness, emphasizing the need for advances in AD diagnostic strategies to better support patients and their families [7]. Current strategies to diagnose AD involve a combination of cognitive assessments, neuroimaging techniques, and the detection of disease-specific indicators, also known as biomarkers [8]. The primary AD biomarkers include two specific molecules: beta-amyloid and tau [9, 10]. Under pathological circumstances, these molecules clump together, and with time, they can accumulate and form into beta-amyloid plaques and neurofibrillary tangles in the brain, resulting in premature death of neural cells, ultimately causing the brains of patients with AD to shrink [11]. The detection of these biomarkers has been increasingly important with the approval of amyloid-targeting treatments, which initiation (and eligibility) often require biomarker confirmation of amyloid pathology [12, 13]. However, there is currently limited empirical evidence from randomized controlled trials demonstrating that earlier diagnosis using biofluid biomarker tests could lead to improved health outcomes compared with later diagnosis [14]. Meanwhile, observation studies have found that AD biomarker testing was associated with decreased health care resource use, therapy optimization, and improved counselling [15].

There are several strategies to detect AD biomarkers. One strategy involves using neuroimaging techniques, such as computed tomography, magnetic resonance imaging, and positron emission tomography (PET), which allows the visualization of abnormal accumulation of beta-amyloid and tau in the brain [16–18]. While neuroimaging techniques are less invasive, they typically detect these biomarkers at later stages of AD, by which point the patient may have already been experiencing cognitive symptoms [19]. Another strategy is to test the concentration of beta-amyloid and tau in biofluid samples, including blood and cerebrospinal fluid (CSF) [8, 20]. These molecules can accumulate as early as 20 years before the onset of cognitive symptoms [17], representing an opportunity for biofluid biomarker tests to diagnose AD in a timely manner [21, 22]. However, CSF sampling requires a lumbar puncture, an invasive procedure that can cause discomfort and add procedural costs [23]. In this regard, blood-based biomarker tests are less invasive, but access may be limited due to variations in healthcare resources [13].

Altogether, the clinical uncertainties, and the differences in invasiveness and availability elevate the importance of evaluating the economic implications of biofluid biomarker testing for diagnosing AD, as their cost-effectiveness may play a vital role in its adoption in clinical practices and translate into significant economic consequences, not only for the patients but also for the healthcare systems. Therefore, evaluating the cost-effectiveness of these tests could be of significant importance for patients and healthcare providers to make informed decisions [23, 24]. Economic evaluation is a key approach in this process by comparing the health benefits, such as improved quality of life and treatment effectiveness, with the costs of diagnostic procedures and follow-up care [25]. These evaluations help to establish whether a diagnostic strategy can be financially viable when implemented in clinical practice. However, differences in the evaluation methods and the factors considered (e.g., demographics and healthcare settings) can significantly influence the results [26]. The existing literature on the economic evaluation of using biofluid biomarker testing to diagnose AD is limited. One systematic review from 2014 assessed the methodological quality of economic evaluations for timely AD diagnostic strategies and found the overall quality was good, but there were insufficiencies in data identification and uncertainty analyses [27]. However, the review was conducted over a decade ago and did not specifically focus on diagnostic strategies through testing biofluid samples to detect AD biomarkers. Given the rapid development of biofluid biomarker testing, the economic landscape has likely shifted, needing a reassessment of cost-effectiveness.

In this systematic review, we examined the cost-effectiveness of biofluid biomarker tests for diagnosing AD in comparison with other diagnostic strategies, including neuroimaging techniques and cognitive assessment. We also evaluated the quality of the economic evaluation conducted, along with their methodology characteristics. Additionally, we identified key contributors that may influence the cost-effectiveness of biofluid biomarker testing as a diagnostic tool for AD.

## Methods

The review followed the Preferred Reporting Items for Systematic Reviews and Meta-analyses (PRISMA) guidelines [28]. The PRISMA checklist is included in Supplementary Table 1. The protocol for this review was registered in the PROSPERO database on November 4th, 2024 (CRD42024596132).

### Search strategy

We conducted a comprehensive electronic search across four databases: PubMed/MEDLINE, Embase, Cochrane Library, and Web of Science, for articles published through October 2024. A research librarian (MB) designed and conducted the search strategy. Specifically, it included MeSH terms for economic evaluations (cost-effectiveness analysis, cost-benefit analysis, cost-utility analysis, cost-saving, return on investment, quality-adjusted life years), biofluid biomarker tests (biomarkers/cerebrospinal fluid, biomarkers/blood, biological marker), and AD (Alzheimer’s Disease, acute confusional senile dementia). The full search strategy is listed in Supplementary Table 2.

### Eligibility criteria

We included studies based on the following criteria:

1. Published in English.
2. Focused on a population that can be both empirical or theoretical that is suffering or suspected of suffering from AD or related dementia.
3. Conducted economic evaluations on biomarker testing from either CSF or blood samples for the diagnosis of AD through methods like cost-effective analysis or cost-utility analysis. The evaluation is conducted based on a healthcare setting, such as memory clinics and primary health clinics.
4. The evaluation included comparisons between the intervention, biomarker testing, and other comparative AD diagnostic strategies, such as neuroimaging techniques (e.g., PET and MRI) and cognitive assessments (e.g., mini-mental state examination).
5. Provided quantifiable economic evaluation outcomes, such as total cost savings, Incremental Cost-effectiveness Ratio (ICER), and Incremental Net Monetary Benefit (INMB). The ICER compares the additional cost of the biomarker testing to its additional health benefits, and the INMB takes one step further by showing whether the benefits are worth the costs based on various willingness-to-pay levels for each unit of health improvement.

We excluded studies if full-text was not available, lacked economic evaluation and/or outcomes, or evaluated the wrong intervention (i.e., not for CSF or blood biomarker testing).

### Data management and screening

The titles and abstracts generated from the search strategy were imported into Rayyan, a web-based software for supporting systematic reviews [29]. After duplicates were removed, two authors (HJ, SE) conducted the initial eligibility screening. The two authors then reviewed the full text of the selected abstracts independently, guided by the inclusion and exclusion criteria above. If the authors disagreed with the decision to include a study, a third reviewer (FS-L) mediated following a discussion with the two authors.

### Data extraction and analysis

A data extraction form was developed to collect information from articles that met the inclusion criteria in Microsoft Excel. The following data was extracted: author, country, year, main objective, economic evaluation method and perspective, study population, health care setting, model type, biomarker testing diagnostic strategies, comparative diagnostic strategies, treatment intervention, cost and benefits, time horizon, sensitivity analyses, and conclusions. We used the narrative approach to synthesize data due to the heterogeneity in study designs, cost perspectives, and healthcare settings. We evaluated methodological quality and characteristics through model design, costs and benefits considerations, as well as sensitivity analyses to comprehensively assess the potential of cost-effectiveness for biofluid biomarker testing. The main economic evaluation outcomes were extracted and organized in a table. To correct the time and currency differences across studies for comparability, all economic values were adjusted to October 2024 real U.S. dollars using the CPI inflation calculator from the U.S. Bureau of Labor Statistics [30] and Currency Exchange Rates Converter from U.S. Treasury Fiscal Data [31].

### Study quality and risk of bias

The quality and risk of bias of the included studies were evaluated using the Consolidated Health Economics Evaluation Reporting Standards (CHEERS) 2022 [32]. The CHEERS checklist consists of 28 items and is a standardized guideline for reporting health economics evaluations. Through CHEERS, the goal was to assess the quality, methodological rigor, and potential biases in economic evaluations of biofluid biomarker testing. Two authors (HJ, SE) assessed whether the included articles sufficiently addressed each of the CHEERS items, with possible responses of “yes” or “no.” We calculated the number of studies that met each criterion (labeled “yes”) and summarized it in a table (supplementary Table 3), along with a qualitative narrative describing how the quality assessment results could affect the review’s findings.

## Results

**Fig. 1:**
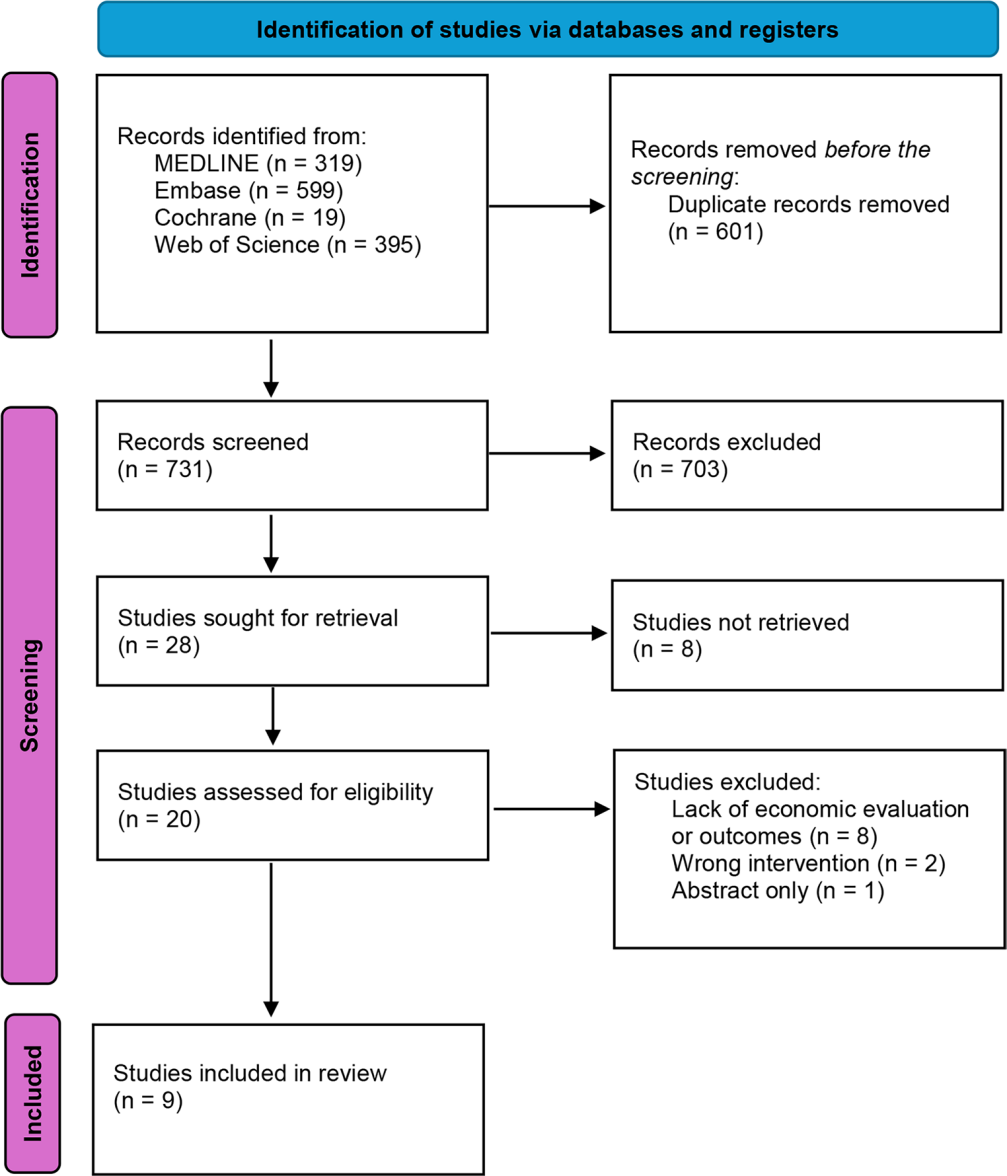
PRISMA flow diagram

The search strategy yielded 731 unique abstracts; 20 were selected for full-text screening. Among these studies, one was excluded due to the lack of full-text availability, eight due to the lack of economic evaluation or outcomes, and two due to the intervention being unrelated to biomarker testing. The remaining nine studies were included in the review (Fig. 1).

### Study characteristics

An overview of study characteristics is summarized in Table 1. Notably, all included studies conducted economic evaluations using mathematical models, and none used data derived from clinical trials. The cost-effectiveness of CSF biomarker tests was evaluated in six studies [4, 33–37], and three studies evaluated blood biomarker tests [38–40]. Comparative diagnostic strategies included physical, clinical, and neuropsychological assessments, either individually or in combination with neuroimaging techniques such as PET.

**Table 1:** Summary of the study characteristics

Study characteristics	Number of studies	References
	(*N* = 9)	
Countries		
Spain	2	^[36, 37]^
Sweden	2	^[33, 38]^
Netherlands	1	^[34]^
US	4	^[4, 35, 39, 40]^
Biofluid Biomarker		
CSF	6	^[4, 33–37]^
Blood	3	^[38–40]^
Comparative strategies		
Neuroimaging	3	^[36, 39, 40]^
Neuropsychological assessment	3	^[33, 35, 37]^
Combined	3	^[4, 34, 38]^
Study population		
Adults (> 65) with MCI	6	^[33–35, 37–39]^
Adults (< 65) with MCI or dementia	1	^[36]^
MCI patients (age not specified)	2	^[4, 40]^
Healthcare Setting		
Memory clinic	4	^[4, 33, 34, 36]^
Memory and primary health clinic	2	^[38, 39]^
Not specified	3	^[35, 37, 40]^

CSF – cerebrospinal fluid; MCI- mild cognitive impairment

The healthcare settings chosen for these models were primarily memory clinics and primary care clinics [4, 33, 34, 36, 38, 39]. The studies were conducted from the perspective of public healthcare systems in Europe and Medicare payers in the U.S [4, 33–39]., with one exception that evaluated the perspective of the patients, focusing on out-of-pocket expenses [40]. The population mainly consisted of older adults with mild cognitive impairments [4, 35, 37–40], and only one study considered adults under 65 years of age suffering from early-onset dementia [36]. Regarding the actions taken upon the test result, some studies incorporated post-diagnosis treatment plans, including referral to specialists in memory clinics and disease-modifying treatment (e.g., donepezil, ChEI) [4, 33–35, 38]. A detailed summary of the included studies can be found in Table 2.

**Table 2:** Summary of included studies (*n* = 9)

Author		Publication year		Objective		Perspective		Model type		Intervention		Comparator		
Aye et al. [38]		2024		Assess the value of blood biomarker testing as part of AD diagnostic workflow		Healthcare system		Decision tree and Markov Model		Blood biomarker test as a guide for referrals and a triage tool for further AD diagnosis		Cognitive tests, structural imaging, and laboratory examinations		
Canestaroet al [39]		2024		Assess the value of blood biomarker testing as part of clinical care pathway		Medicare		Decision tree and budget impact model		Blood biomarker as a screening tool to diagnose AD		Usual care testing with PET biomarker test or CSF		
Contador et al. [36]		2023		Evaluate the cost-effectiveness of CSF biomarker tests		Healthcare system		Decision tree		CSF biomarker test to diagnose AD		Amyloid PET		
Handels et al. (2015) [34]		2015		Assess the economic value of CSF biomarker tests		Healthcare system		Decision tree		CSF as an add-on test to current clinical practice		Physical, clinical, and neuropsychological examination and MRI		
Handels et al. (2017) [33]		2017		Estimate the cost-effectiveness CSF biomarker testing to standard diagnostic workup		Healthcare system		Decision tree		CSF added to the usual care for AD prognosis		Diagnostic test workup in usual care		
Michaud et al. [35]		2017		Assess the cost effectiveness of CSF biomarkers as a risk-stratification tool		Medicare		Markov model		Test- treat strategies based on the CSF results and to determine three risk levels to AD		Diagnostic tool without CSF		
Lee et al. [4]		2017		Evaluated the cost-effectiveness of CSF biomarker testing		Medicare		Markov model		CSF to conduct biomarker analysis		Clinical assessment and MRI		
Nazco et al. [37]		2014		Determine the cost-effectiveness of CSF to diagnose AD		Healthcare system		Decision tree model		CSF to detect biomarkers of AD		Established AD clinical criteria		
Noda et al. [40]		2024		Examine the cost-effectiveness of blood biomarker tests		Patients		Decision tree model		Blood tests to detect AD biomarkers		Amyloid PET, CSF		
Author	Post-diagnostic Intervention				Cost categories		Benefit/effect measures		Outcome measure		Sensitivity Analysis Methods		Time Horizon	Conclusion
Aye et al. [38]	Referral to specialist in memory clinics and disease-modifying treatment (27% progression reduction)				Diagnostic, disease-specific costs		Patient Health utilities values (QALY), caregiver values		ICER		PSA and deterministic		30 years	Integration of blood biomarker test can be cost-effective by enabling earlier, more accurate diagnoses
Canestaro et al. [39]	NA				Diagnostic costs		Diagnostic accuracy		Incremental cost per case identified, test accuracy		PSA		1 year period only	Blood biomarker tests offered significant cost savings from the diagnosis process
Contador et al. [36]	NA				Direct healthcare costs (diagnostic and medical visits)		Diagnostic accuracy		ICER		PSA and deterministic		3 months	CSF can be cost-effective 50% of the times
Handels et al. [34]	Hypothetical disease modifying treatment (50% progression reduction)				Diagnostic costs		QALY, utilities reflect the desirability of a health state		Lifetime average cost, INMB		PSA and deterministic		Lifetime	More potential benefits from CSF that was used to identify missed AD cases
Handels et al. [33]	Follow-up visits				Diagnostic, disease-specific costs		QALY, impact of the prognosis and quality of life		Average INMB, incremental cost		PSA and deterministic		5 years	CSF is cost-effective in pre-dementia stage but sensitive to input parameters
Michaud et al. [35]	ChEI treatment (more effective for mild AD patients)				Disease-specific, informal care, medical and pharmaceutical costs		QALY, health related quality of life		ICER		PSA and deterministic		Lifetime	CSF for early targeted treatment among patients with mild cognitive impairments may be cost-effective
Lee et al. [4]	Donepezil in mild and moderate AD; memantine in severe AD				Diagnostic, AD-specific, location supportive care costs		QALY, AD-specific quality of life		lifetime discounted costs, ICER		PSA and deterministic		Lifetime	CSF is cost-effective when AD prevalence is at least 9%
Nazco et al. [37]	Follow-up visits and treatment with donepezil				Direct health care costs		Diagnostic accuracy		ICER		PSA and deterministic		Lifetime	CSF as biomarkers is a cost-effective but with high uncertainty
Noda et al. [40]	NA				Diagnostic costs (out of pocket expenses)		Diagnostic accuracy		ICER		PSA and deterministic		N/A	Potential value for blood biomarker tests to improve AD test specificity

INMB – incremental net monetary benefit; ICER – incremental cost-effective ratios; PSA – probabilistic sensitivity analysis; QALY- quality-adjusted life years; CSF – cerebrospinal fluid; PET – positron emission tomography; AD – Alzheimer’s Disease

### Economic evaluation models

**Fig. 2:**
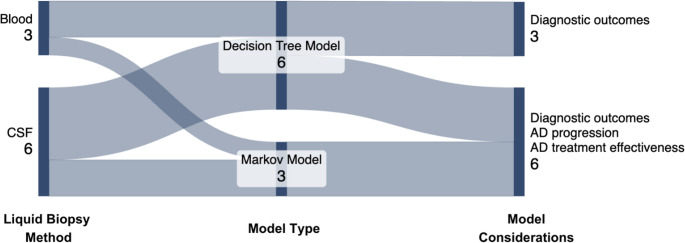
Sankey diagram of economic evaluation model type and considerations

While all studies conducted model-based analysis, the model design and considerations were not always the same (Fig. 2). The most popular design was the decision tree model, utilized in six studies [33, 34, 36, 37, 39, 40]. These models visually represent the decision-making process by originating from a single starting point, in this case, the diagnostic strategy, and then analyzing the potential consequences based on the diagnostic outcomes, namely, true/false positive and true/false negative diagnoses. Among these six studies, three focused on evaluating the short-term outcomes, ending with the diagnosis of AD [36, 39, 40]. The other three studies extended the evaluation to also consider AD treatment and disease progression post-diagnosis of AD [33, 34, 37]. These extended evaluations incorporated factors such as the influence of the diagnostic accuracy on treatment outcomes, including quality-of-life improvements. They also modeled annual cognitive decline and a score to assess patients’ abilities to manage their physical needs and daily living activities to simulate the progression of AD [33, 34].

The remaining three studies used the Markov model design [4, 35, 38]. In Markov models, patients are categorized into different health states based on AD disease severity (i.e., mild → moderate → severe) and residential settings (community care → long-term care facilities). These health states are not permanent, and patients can transition periodically (annually [35, 38] or monthly [4]) into more advanced disease states, ultimately ending in estimated mortality. The rates at which patients transitioned from one health state to another were also modeled to assess treatment effectiveness and disease progression based on the timing and accuracy of the diagnosis. For instance, patients with mild (early stages) AD who were accurately diagnosed and treated are modeled with a slower transition to more severe stages, which reflects a better effectiveness of the selected treatment [35]. The Markov models in the included studies also had a longer time horizon compared to the decision tree models, covering a period from 30 years to a lifetime.

### Costs

The costs were estimated using previous literature and considering each country’s healthcare context. The direct medical costs were considered in all studies, including expenses associated with lumbar puncture for CSF samples and venipuncture for blood tests, as well as the fees for neuroimaging scans and costs related to follow-up visits. Four studies considered the AD treatment costs, which included prescription expenses throughout the treatment periods [34, 35, 37, 38]. The disease-specific costs associated with each stage of AD and residential setting were estimated in four studies [4, 35, 36, 38]. Only one study considered indirect healthcare costs, including paid and unpaid caregiving [4].

### Benefits

Five of the nine selected studies measured quality-adjusted life years (QALY) to quantify the benefits the patients may gain from the various AD diagnostic strategies [4, 33–35, 38]. QALY reflects both patients’ quantity and quality of life, with values ranging from 0 (death) to a maximum of 1 (one year in perfect health). Specifically, three of these studies relied on standard health status measurements such as the EuroQol-5D [34, 38] and Health Utilities Index Mark II to estimate QALYs [35], while other studies estimated QALY based on AD severity and diagnostic accuracy [4, 33]. To reflect the transitory impact on quality of life derived from the diagnostic method, QALYs were also used to reflect the discomfort associated with the lumbar puncture for CSF (0.008–0.01 QALY deduction) [4, 35] and potential AD treatment side effects (0.05 QALY deduction) [34]. In addition, one study also used QALY to consider the emotional values of the patients, including worrying about developing AD and the associated stigma from a false diagnosis [33].

The four remaining studies did not utilize QALY. Instead, health benefits were evaluated through the percentages of correctly diagnosed AD associated with each diagnostic strategy [36, 37, 39, 40]. Unfortunately, the effect of the diagnosis on the quality of life of the patients was not factored in these evaluation models, potentially underestimating the broader health benefits of AD diagnostic approaches.

### Test-treat pathway

To estimate the effect of changes in the diagnostic accuracy of the biofluid biomarker tests on the subsequent treatment decisions, six of the nine selected studies included a simulation of the test-treat pathway in their models. To this end, they considered the influence of the diagnostic accuracy on the treatment decisions, treatment outcomes, and effectiveness [4, 33–35, 37, 38]. Of them, three studies used hypothetical assumptions based on prior literature to model treatment effectiveness [4, 34, 38]. Specifically, one study evaluated the cost-effectiveness of treatment based on two intervention strategies: “verify AD” or “rule out AD”, and treatment effects were hypothetically assumed to be a constant 50% reduction in progression across all patients [34]. In another study, the authors incorporated data from clinical trials evaluating the efficacy of two treatments (aducanumab and lecanemab), and modeled a 27% reduction in disease progression and assumed a 100% treatment response among all patients with a positive diagnosis [38]. Lastly, one study considered the benefit of treatment (memantine), estimating an improved quality of life for patients with severe AD (0.051 QALYs per year) [4].

Among the remaining three studies that consider the test-treat pathway, one did not include treatment effectiveness, as they stated there was no prior empirical evidence on its effectiveness for patients with mild cognitive impairment [33]. Instead, their treatment decisions from improvement in test accuracy were reflected through frequency and duration of follow-up care, as well as the effect of stigmatization and worrying associated with a false positive and negative diagnosis. Another study used six test-treat strategies (from no testing and treating all AD patients, to testing and treating patients with low risk of developing AD) to accommodate variability in patients’ conditions [35]. Very different from the rest, one study used the costs associated with treatments and associated follow-up care to simulate the consequences of the diagnostic outcomes [37].

### Sensitivity analysis

To consider the inherent uncertainties in model-based evaluations, all studies conducted a form of sensitivity analysis to access the influence of variations in key input parameters (e.g., diagnostic test accuracy, AD prevalence) on the cost-effectiveness of the different methods to diagnose AD. To do so, all studies used probabilistic sensitivity analysis and Monte Carlo simulations. These methods involved running the models thousands of times with randomly selected parameter values based on their probability distributions. The results were then compared against a willingness-to-pay threshold to calculate the probability of cost-effective biomarker testing based on how often results fell below this threshold. Additionally, eight studies conducted a one-way deterministic sensitivity analysis alongside the probabilistic sensitivity analysis, adjusting one parameter at a time to assess the effect on model outcomes [4, 33–38, 40].

### Economic evaluation outcomes

All studies found that biofluid biomarker testing has the potential to be cost-effective compared with other comparative testing strategies. However, the probability and the magnitude of cost-effectiveness varied significantly. The primary outcomes from the economic evaluations are summarized in Table 3.

**Table 3:** Economic evaluation outcomes

Author	Net outcomes	Net costs per patient ($ in 2024)	Measurement	Value ($ in 2024)	Probability of cost-effectiveness (WTP)
Aye et al. [38]	0.01 QALY	$640	ICER	$52,099	48% (€50,000)
Canestaro et al. [39]	−0.43% sensitivity - 0.51% specificity	-$643	Cost savings	$3.57 million cost savings (11.4% reduction in cost)	NA
Contador et al. [36]	−7.4% correct diagnosis	-$1641	ICER	$21,722	50% (€19,840)
Handels et al. [34]	0.39 QALY	-$48,147	Incremental net monetary benefit *	$108,896	NA
Handels et al. [33]	0.046 QALY	$595	ICER	$16,595	40% (€20,000) 6% (€80,000)
Michaud et al. [35]	0.024 QALY	$1,463	ICER	$48,190	26% ($100,000) 29% ($150,000)
Lee et al. [4]	0.015 QALY	$214	ICER	$14,101	72% ($50,000) 82% ($100,000)
Nazco et al. [37]	36% accurate diagnosis	-$2,622	Cost savings	$2622/patient	Dominant
Noda et al. [40]	−10.88% accurate diagnosis	$3805.3 (total cost)	incremental cost per case identified, test accuracy	$349.89 per 1% increase in test accuracy	< 10% ($1,000)

* The study assumed a hypothetical perfect CSF test (100% accuracy)

ICER – incremental cost-effectiveness ratio; WTP – willingness-to-pay

Half of the six studies evaluating biofluid biomarker testing via CSF for diagnosing AD concluded that CSF offered greater health benefits, albeit having a higher cost than alternative testing strategies. These studies reported the incremental cost-effective ratio (ICER) per QALY, representing the cost of gaining one year of perfect health through CSF to diagnose AD. The result ranged from $16,595 to $48,190 ($ in 2024) [4, 33, 35]. CSF biomarker tests were concluded to be cost-effective in these studies, as the willingness-to-pay thresholds were higher than the ICERs, meaning the additional costs were justified by the health benefits. Sensitivity analyses revealed that the probability of CSF being cost-effective for AD diagnosis ranged from 26% to 82%, depending on willingness-to-pay thresholds; with higher WTP, CSF biomarker tests showed higher certainty in cost-effectiveness. Additionally, one study found CSF to be the dominant AD diagnostic strategy, meaning it has lower costs and greater health benefits [37]. Another study estimated CSF could lead to a net incremental monetary benefit of $108,896 ($ in 2024) per patient; however, the study assumed a hypothetical perfect CSF test with 100% accuracy. In another study, CSF was shown to be less costly and less effective than amyloid PET. Consequently, the probability of CSF being cost-effective was only at 50% [36].

Economic outcomes from testing blood biomarkers showed mixed results. In one study, implementing a blood biomarker test as a decision tool increased the overall costs but yielded only a slight increase in QALY (0.01). As a result, the ICER was relatively high, reaching $52,099 ($ in 2024) [38]. It was also indicated in the same study that blood biomarker tests were cost-effective less than half the time, highlighting high uncertainty. In another study, blood biomarker tests led to a decrease in the number of patients needing further testing through CSF or PET to confirm AD diagnosis. This was a large driver for an 11.4% cost reduction, which led to a total savings of $3.57 million for 1 million hypothetical patients. However, no significant diagnostic improvement was observed, and the probability of cost-effectiveness was not evaluated [39]. A third study found that blood biomarker tests had lower costs than PET and CSF and lower AD diagnostic accuracy, showing low probability of being cost-effective (< 10%) [40].

### Cost-effectiveness contributors

Through sensitivity analysis, studies identified several key contributors that can significantly influence the cost-effectiveness of biofluid biomarker testing for AD diagnosis. For instance, AD prevalence was found as a key contributor in three studies [4, 34, 35]. Specifically, in one study, CSF biomarker tests were found to be cost-effective when AD prevalence was higher than 9%. Although this threshold varied with age, decreasing to 8% for younger patients and rising to 27% for patients over 75 years old [4]. Another study found that CSF was more likely to be cost-effective for patients at lower risk of progressing to AD [35], while a third study revealed that high testing accuracy was essential to cost-effectiveness in populations with high AD prevalence [34]. These findings suggested that CSF biomarker tests were particularly cost-effective for younger populations with lower AD prevalence rates, highlighting the value of timely AD diagnosis in managing AD.

Another key contributor identified was the sensitivity and specificity of the test used with biofluid biomarkers for diagnosing AD. Six studies found specificity had a significant influence on the cost-effectiveness of biomarker testing [33, 34, 36–38, 40], and sensitivity was only significant in one study. This could potentially be due to the costs of false positive cases (related to specificity), such as unnecessary treatments, being greater than those of false negatives (related to sensitivity). The costs of false negatives were also more difficult to quantify, especially when they were related to delayed treatment and reduced effectiveness. False negatives were also often inadequately captured in models. For example, in one study, avoiding a false positive diagnosis was valued at $11,345, compared to $9,954 for a false negative diagnosis [4]. The one study that found sensitivity to be a significant contributor assessed the psychological benefits of reducing anxiety and stigma associated with false negative diagnoses [33].

Treatment-related factors also contributed significantly to the cost-effectiveness of biofluid biomarker testing. In two studies, variation in the treatment effectiveness had the greatest influence on the ICER of biomarker testing and their cost-effectiveness [35, 38]. Additionally, one study revealed that high treatment adherence rates resulted in an increase in excessive costs for patients who received a false-positive diagnosis, contributing to a lower probability of cost-effectiveness [4].

### Quality assessment

The quality assessment results using the CHEERS 2022 are summarized in Supplementary Table 3. No studies met al.l 28 criteria; the average number of criteria met was 23. Only one study met criterion 20, which described the distribution effects across population groups [4]. No study met the criteria of including approaches to engagement and its effects on patients and others affected by the study. All studies met the criteria related to economic evaluation, including time horizon, perspectives, rational and model design, as well as outcome measurements and uncertainty considerations.

## Discussion

The recent development in biofluid biomarker testing has significantly improved the accuracy of AD diagnosis, particularly in early stages, providing potential economic value for patients and healthcare systems. In this review, we found that CSF biomarker testing is more cost-effective than alternative methods, with its higher costs justified by the health benefits it brings. In contrast, blood-based tests showed mixed results, with some studies suggesting substantial cost savings and others finding a very low probability of cost-effectiveness.

The economic nature of AD is complex, involving the fluctuating progression of AD, diagnosis outcomes, and variations in treatment decisions over long periods. This broad scope can pose challenges to the design and execution of clinical trials, limiting the feasibility of trial-based economic evaluation to assess cost-effectiveness [41]. Such a limitation is reflected in this review as all included studies conducted model-based economic evaluations. However, these models not only showcased the capacity to estimate the cost-effectiveness of diagnostic strategies but also provided tools to investigate how cost-effectiveness will be influenced by the variance of key contributors, simulating the complex reality of AD. Additionally, model-based simulations have been widely used to evaluate new health interventions and support decision-making when empirical data is unavailable. Some examples included the assessment of COVID-19 treatment plans under pandemic circumstances [42], the estimation of the optimal screening age for colorectal cancer [43], and the application of digital preventative public health interventions [44].

The studies reviewed here showed that the decision tree and Markov models offer valuable insights into the cost-effectiveness of different diagnostic strategies for AD. The decision tree model represented a more straightforward approach that offers an easy interpretation and is more frequently used for simulating short-term diagnostic outcomes derived from biofluid biomarker testing. This is evident in studies that reported economic benefits based solely on improved diagnostic accuracy. In contrast, the Markov model provides a more dynamic simulation of the possible consequences of AD diagnosis and is more effective in capturing the long-term effects of biofluid biomarker testing. It not only considers diagnostic accuracy but also integrates other important factors, such as treatment effectiveness and disease progression through various stages of AD. With these additional factors considered, the economic evaluations allowed the assessment of more potential benefits in each diagnostic strategy. Considering that the best practices for model-based economic evaluation of diagnostic tests recommended exploring the indirect benefits of the diagnostic tests [26], the Markov model may be a better approach to evaluate the economic outcomes of using biofluid biomarkers. Our analysis identified that there are contributors, including AD prevalence, diagnostic accuracy, and treatment effectiveness, that significantly influence the cost-effectiveness of biomarker tests. Small variations in these contributors can lead to significant changes in the result of the cost-effectiveness. For instance, low specificity can result in more false positive diagnoses, which can lead to unnecessary treatment costs. Similarly, low sensitivity can result in more false negative diagnoses, leading to delayed treatment and reduced effectiveness. However, the consequences of these inaccurate diagnoses were overlooked or insufficiently modeled in some studies. For instance, studies that largely focused on the percentage of correctly diagnosed AD as the primary measurement of health benefits often failed to account for the costs and diminished quality of life associated with delayed diagnosis and treatment, potentially underestimating the consequences of false negatives. Similar challenges were also identified in other economic evaluation reviews [45].

Modeling the economic consequences of biofluid biomarker testing for AD patients is challenging, as illustrated by the different methodological approaches used to incorporate test-treat pathways analysis into the models across studies. Notwithstanding, for AD patients, the consideration of test-treat pathways is particularly important as the diagnostic outcomes directly influence treatment choice, timing, and effectiveness in delaying the progression of AD [46]. While most studies in this review included some form of the test-treat pathway in their models, many relied on hypothetical assumptions and uniform response across all patients (i.e., assuming all patients with a positive test will receive treatment and have a response). Such a simplified assumption may limit its applicability to real-life clinical practices, with the potential consequence of overestimating the benefits associated with improved diagnostic accuracy. This is often due to patients with a true positive diagnostic outcome being modeled to receive and respond to treatment, which is unlikely to be the case for AD management, where treatment uptake and response are known to be uncertain and heterogeneous [47, 48]. Good practice recommendations for modeling the cost-effectiveness of diagnostic tests pointed out that, although there is currently no specific methodological guidance on how to model the test-treat pathway, it is important to consider the probabilities based on clinical practices regarding the percentage of patients with positive diagnoses who actually receive treatment, and to account for the various strategies based on clinical and patient factors [49]. One example of how to implement this recommendation was found in one of the reviewed studies. This study modeled six potential test–treat strategies, ranging from universal testing and treatment to selective testing and treatment based on risk profiles, thereby simulating the realistic diversity of test-treat pathways for AD patients [35].

Our findings also suggest that incorporating patient-reported outcomes in economic evaluations has the potential to improve the measurement of health benefits [50]. However, even though AD is a deeply emotional disease that profoundly changes the lives of patients and their families, it was intriguing to find a lack of emphasis on patient-reported outcomes and quality-of-life measures in the studies reviewed. While some studies used generic measures, such as EuroQol-5D, to estimate QALY, these may not reflect the nuanced burden associated with AD, including anxiety and stress. Only one study included emotional factors such as worrying and stigma associated with AD diagnostic outcomes.

Our systematic review is not without limitations. All included studies conducted the analysis based on the healthcare environments of Europe and the U.S., which limits the implications of the findings to other regions of the world. Given the diversity in healthcare resources, the cost-effectiveness of biofluid biomarker testing could differ significantly. Second, we included only nine studies, which were all using model-based economic evaluations with heterogeneous designs, making it difficult to generalize the results. Future evaluations on biofluid biomarker testing for AD diagnosis should also consider patients’ quality of life to attempt a more realistic reflection of the health benefits and costs associated with diagnostic outcomes. Additionally, as recommended by CHEERS, engaging patients and other key individuals, such as family members and clinicians, during the evaluation process may improve the quality and rigor of the economic evaluation.

## Conclusion

Our review suggests that biofluid biomarker testing may have the potential to be a significant cost-effective strategy for the timely diagnosis of AD. This is particularly true for CSF biomarker tests, where improved diagnostic accuracy for AD could support timely management of the disease, though this depends on assumptions for treatment effectiveness, which influence the estimated benefits from accurate AD diagnosis. While blood biomarker testing showed mixed results in cost-effectiveness, it demonstrated that it is a screening tool that can result in cost savings. Our findings underscore the importance of incorporating the impact of diagnostic strategies on the AD patient’s quality of life when evaluating their cost-effectiveness. Model-based analyses have provided valuable information, and we hope future economic evaluations will incorporate more real-world data from clinical trials. The assumptions related to treatment post-diagnosis require cautious interpretation of the results from this review, which may offer preliminary guidance for patients and healthcare systems on making informed decisions on choosing AD diagnostic strategies.

## Supplementary Information

Below is the link to the electronic supplementary material.

Supplementary File 1 (DOCX 269 KB)

Supplementary File 2 (DOCX 18.6 KB)

Supplementary File 3 (DOCX 22.9 KB)

## Data Availability

All relevant data is available in the manuscript and supplementary materials.

## References

[1] Gustavsson, A., et al.: Global estimates on the number of persons across the alzheimer’s disease continuum, (in eng). Alzheimers Dement. **19**(2), 658–670 (2023). 10.1002/alz.12694 10.1002/alz.12694 35652476

[2] Niu, H., Álvarez-Álvarez, I., Guillén-Grima, F., Aguinaga-Ontoso, I.: Prevalence and incidence of Alzheimer’s disease in Europe: A meta-analysis, (in eng|spa). Neurologia*. ***32**(8), 523–532 (2017). 10.1016/j.nrl.2016.02.016 10.1016/j.nrl.2016.02.016 27130306

[3] England, K., Azzopardi-Muscat, N.: Demographic trends and public health in Europe, (in eng). Eur. J. Public Health. **27**(4), 9–13 (2017). 10.1093/eurpub/ckx159 10.1093/eurpub/ckx159 29028238

[4] Lee, S.A., Sposato, L.A., Hachinski, V., Cipriano, L.E.: Cost-effectiveness of cerebrospinal biomarkers for the diagnosis of Alzheimer’s disease, (in eng). Alzheimers Res. Ther*. ***9**(1), 18-16 (2017). 10.1186/s13195-017-0243-0 10.1186/s13195-017-0243-0 PMC5356269 28302164

[5] Atri, A.: The Alzheimer’s Disease Clinical Spectrum: Diagnosis and Management, (in eng). Med.Clin. North Am. **103**(2), 263–293 (2019). 10.1016/j.mcna.2018.10.009 10.1016/j.mcna.2018.10.009 30704681

[6] Liss, J.L., et al.: Practical recommendations for timely, accurate diagnosis of symptomatic Alzheimer’s disease (MCI and dementia) in primary care: a review and synthesis, (in eng). J. Intern Med*.* **290**(2), 310–334 (2021). 10.1111/joim.13244 10.1111/joim.13244 PMC8359937 33458891

[7] Geldmacher, D.S., et al.: Implications of early treatment among Medicaid patients with Alzheimer’s disease, (in eng). Alzheimers Dement. **10**(2), 214 – 24 (2014). 10.1016/j.jalz.2013.01.015 10.1016/j.jalz.2013.01.015 23643457

[8] Juganavar, A., Joshi, A., Shegekar, T.: Navigating early alzheimer’s diagnosis: A comprehensive review of diagnostic innovations, (in eng). Cureus. **15**(9), e44937 (2023). 10.7759/cureus.44937 10.7759/cureus.44937 PMC10561010 37818489

[9] Porsteinsson, A.P., Isaacson, R.S., Knox, S., Sabbagh, M.N., Rubino, I.: Diagnosis of early alzheimer’s disease: clinical practice in 2021. The Journal of Prevention of Alzheimer’s Disease **8**(3), 371–386 (2021). 10.14283/jpad.2021.23 34101796 10.14283/jpad.2021.23 PMC12280795

[10] Jack, C.R., et al.: Revised criteria for diagnosis and staging of alzheimer’s disease: alzheimer’s association workgroup, (in eng). Alzheimers Dement. **20**(8), 5143–5169 (2024). 10.1002/alz.13859 10.1002/alz.13859 PMC11350039 38934362

[11] Escamilla, S., et al.: Synaptic and extrasynaptic distribution of NMDA receptors in the cortex of Alzheimer’s disease patients,. Alzheimer’s & Dementia **20**(12), 8231–8245 (2024). 10.1002/alz.14125 10.1002/alz.14125 PMC11667538 39450669

[12] Rosen, J., Jessen, F.: Patient eligibility for amyloid-targeting immunotherapies in Alzheimer’s disease,. The Journal of Prevention of Alzheimer’s Disease **12**(4), 100102 (2025). 10.1016/j.tjpad.2025.100102 40011174 10.1016/j.tjpad.2025.100102 PMC12184045

[13] Schindler, S.E., et al.: Acceptable performance of blood biomarker tests of amyloid pathology - recommendations from the Global CEO Initiative on Alzheimer’s Disease,. Nat Rev Neurol **20**(7), 426–439 (2024). 10.1038/s41582-024-00977-5 38866966 10.1038/s41582-024-00977-5

[14] Kokkinou, M., et al.: Plasma and cerebrospinal fluid ABeta42 for the differential diagnosis of Alzheimer’s disease dementia in participants diagnosed with any dementia subtype in a specialist care setting,. Cochrane Database Syst Rev **2**(2), CD010945 (2021). 10.1002/14651858.CD010945.pub2 33566374 10.1002/14651858.CD010945.pub2 PMC8078224

[15] Patel, K.J., et al.: Clinical value of Alzheimer’s disease biomarker testing,. Alzheimer’s & Dementia: Translational Research & Clinical Interventions **10**(2), e12464 (2024). 10.1002/trc2.12464 38596484 10.1002/trc2.12464 PMC10999950

[16] Veitch, D.P., et al.: Using the Alzheimer’s disease neuroimaging initiative to improve early detection, diagnosis, and treatment of Alzheimer’s disease,. Alzheimers Dement **18**(4), 824–857 (2022). 10.1002/alz.12422 34581485 10.1002/alz.12422 PMC9158456

[17] Escamilla, S., Salas-Lucia, F.: Thyroid hormone and alzheimer disease: bridging epidemiology to mechanism,. Endocrinology (2024). 10.1210/endocr/bqae124 39276028 10.1210/endocr/bqae124

[18] Salas-Lucia, F.: Mapping thyroid hormone action in the human brain,. Thyroid **34**(7), 815–826 (2024). 10.1089/thy.2024.0120 38757586 10.1089/thy.2024.0120 PMC11295854

[19] Boerwinkle, A.H., et al.: Temporal Correlation of CSF and Neuroimaging in the Amyloid-Tau-Neurodegeneration Model of Alzheimer Disease,. Neurology **97**(1), e76–e87 (2021). 10.1212/WNL.0000000000012123 33931538 10.1212/WNL.0000000000012123 PMC8312859

[20] Olsson, B., et al.: CSF and blood biomarkers for the diagnosis of Alzheimer’s disease: a systematic review and meta-analysis, (in eng). Lancet Neurol. **15**(7), 673–684 (2016). 10.1016/S1474-4422(16)00070-3 10.1016/S1474-4422(16)00070-3 27068280

[21] Caselli, R.J., et al.: Neuropsychological decline up to 20 years before incident mild cognitive impairment, (in eng). Alzheimers Dement. **16**(3), 512–523 (2020). 10.1016/j.jalz.2019.09.085 10.1016/j.jalz.2019.09.085 PMC7067658 31787561

[22] Ausó, E., Gómez-Vicente, V., Esquiva, G.: Biomarkers for Alzheimer’s disease early diagnosis, (in eng). J. Pers. Med. **10**(3), (2020). 10.3390/jpm10030114 10.3390/jpm10030114 PMC7563965 32899797

[23] Khan, T.K., Alkon, D.L.: Alzheimer’s Disease Cerebrospinal Fluid and Neuroimaging Biomarkers: Diagnostic Accuracy and Relationship to Drug Efficacy. J Alzheimers Dis **46**(4), 817–36 (2015). 10.3233/JAD-150238 26402622 10.3233/JAD-150238

[24] Nandi, A., et al.: Cost of care for Alzheimer’s disease and related dementias in the United States: 2016 to 2060, (in eng). NPJ Aging. **10**(1), 13 (2024). 10.1038/s41514-024-00136-6 10.1038/s41514-024-00136-6 PMC10853249 38331952

[25] Snowsill, T.: Modelling the cost-effectiveness of diagnostic tests, (in eng). Pharmacoeconomics. **41**(4), 339–351 (2023). 10.1007/s40273-023-01241-2 10.1007/s40273-023-01241-2 36689124

[26] Shinkins, B., Yang, Y., Abel, L., Fanshawe, T.R.: Evidence synthesis to inform model-based cost-effectiveness evaluations of diagnostic tests: A methodological review of health technology assessments, (in eng). BMC Med. Res. Methodol. **17**(1), 56 (2017). 10.1186/s12874-017-0331-7 10.1186/s12874-017-0331-7 PMC5391551 28410588

[27] Handels, R.L., Wolfs, C.A., Aalten, P., Joore, M.A., Verhey, F.R., Severens, J.L.: Diagnosing Alzheimer’s disease: a systematic review of economic evaluations, (in eng). Alzheimers Dement*. ***10**(2), 225 – 37 (2014). 10.1016/j.jalz.2013.02.005 10.1016/j.jalz.2013.02.005 23727080

[28] Page, M.J., et al.: The PRISMA 2020 statement: an updated guideline for reporting systematic reviews, (in eng). BMJ. **372**, n71 (2021). 10.1136/bmj.n71 10.1136/bmj.n71 PMC8005924 33782057

[29] Ouzzani, M., Hammady, H., Fedorowicz, Z., Elmagarmid, A.: Rayyan-a web and mobile app for systematic reviews, (in eng). Syst. Rev. **5**(1), 210 (2016). 10.1186/s13643-016-0384-4 10.1186/s13643-016-0384-4 PMC5139140 27919275

[30] CPI Inflation Calculator. U.S. Bureau of Labor Statistics: (2023). https://www.bls.gov/data/inflation_calculator.htm

[31] Data, U.S.T.F.: Currency exchange rates converter, ed

[32] Husereau, D., et al.: Consolidated Health Economic Evaluation Reporting Standards 2022 (CHEERS 2022) Statement: Updated Reporting Guidance for Health Economic Evaluations. Value Health **25**(1), 3–9 (2022). 10.1016/j.jval.2021.11.1351 35031096 10.1016/j.jval.2021.11.1351

[33] Handels, R.L.H., et al.: Cost-Utility of Using Alzheimer’s Disease Biomarkers in Cerebrospinal Fluid to Predict Progression from Mild Cognitive Impairment to Dementia. J Alzheimers Dis **60**(4), 1477–1487 (2017). 10.3233/JAD-170324 29081416 10.3233/JAD-170324

[34] Handels, R.L., et al.: Early cost-utility analysis of general and cerebrospinal fluid-specific Alzheimer’s disease biomarkers for hypothetical disease-modifying treatment decision in mild cognitive impairment. Alzheimer’s & Dementia **11**(8), 896–905 (2015). 10.1016/j.jalz.2015.02.009 10.1016/j.jalz.2015.02.009 26071009

[35] Michaud, T.L., Kane, R.L., McCarten, J.R., Gaugler, J.E., Nyman, J.A., Kuntz, K.M.: Using Cerebrospinal Fluid Biomarker Testing to Target Treatment to Patients with Mild Cognitive Impairment: A Cost-Effectiveness Analysis. PharmacoEconomics - Open **2**(3), 309–323 (2018). 10.1007/s41669-017-0054-z 29623628 10.1007/s41669-017-0054-z PMC6103924

[36] Contador, J., Vargas-Martínez, A.M., Sánchez-Valle, R., Trapero-Bertran, M., Lladó, A.: Cost-effectiveness of Alzheimer’s disease CSF biomarkers and amyloid-PET in early-onset cognitive impairment diagnosis. Eur Arch Psychiatry Clin Neurosci **273**(1), 243–252 (2023). 10.1007/s00406-022-01439-z 35710952 10.1007/s00406-022-01439-z

[37] Valcárcel-Nazco, C., Perestelo-Pérez, L., Molinuevo, J.L., Mar, J., Castilla, I., Serrano-Aguilar, P.: Cost-effectiveness of the use of biomarkers in cerebrospinal fluid for Alzheimer’s disease. J Alzheimers Dis **42**(3), 777–88 (2014). 10.3233/JAD-132216 24916543 10.3233/JAD-132216

[38] Aye, S., Handels, R., Winblad, B., Jönsson, L.: Optimising Alzheimer’s Disease Diagnosis and Treatment: Assessing Cost-Utility of Integrating Blood Biomarkers in Clinical Practice for Disease-Modifying Treatment. The Journal of Prevention of Alzheimer’s Disease **11**(4), 928–942 (2024). 10.14283/jpad.2024.67 39044504 10.14283/jpad.2024.67 PMC11266371

[39] Canestaro, W.J., Bateman, R.J., Holtzman, D.M., Monane, M., Braunstein, J.B.: Use of a Blood Biomarker Test Improves Economic Utility in the Evaluation of Older Patients Presenting with Cognitive Impairment. Population Health Management **27**(3), 174–184 (2024). 10.1089/pop.2023.0309 38546435 10.1089/pop.2023.0309 PMC11304753

[40] Noda, K., Lim, Y., Goto, R., Sengoku, S., Kodama, K.: Cost-effectiveness comparison between blood biomarkers and conventional tests in Alzheimer’s disease diagnosis. Drug Discov Today **29**(3), 103911 (2024). 10.1016/j.drudis.2024.103911 38311028 10.1016/j.drudis.2024.103911

[41] Abbott, J.H., Wilson, R., Pryymachenko, Y., Sharma, S., Pathak, A., Chua, J.Y.Y.: Economic evaluation: a reader’s guide to studies of cost-effectiveness. Arch Physiother **12**(1), 28 (2022). 10.1186/s40945-022-00154-1 36517825 10.1186/s40945-022-00154-1 PMC9753355

[42] Veijer, C., van Hulst, M.H., Friedrichson, B., Postma, M.J., van Asselt, A.D.I.: Lessons Learned from Model-based Economic Evaluations of COVID-19 Drug Treatments Under Pandemic Circumstances: Results from a Systematic Review. Pharmacoeconomics **42**(6), 633–647 (2024). 10.1007/s40273-024-01375-x 38727991 10.1007/s40273-024-01375-x PMC11126513

[43] Ramos, M.C., Passone, J.A.L., Lopes, A.C.F., Safatle-Ribeiro, A.V., Ribeiro Júnior, U., de Soárez, P.C.: Economic evaluations of colorectal cancer screening: A systematic review and quality assessment. Clinics (Sao Paulo) **78**, 100203 (2023). 10.1016/j.clinsp.2023.100203 37099816 10.1016/j.clinsp.2023.100203 PMC10182269

[44] Lange, O.: Health economic evaluation of preventive digital public health interventions using decision-analytic modelling: a systematized review. BMC Health Serv Res **23**(1), 268 (2023). 10.1186/s12913-023-09280-3 36932436 10.1186/s12913-023-09280-3 PMC10024449

[45] Iragorri, N., Spackman, E.: Assessing the value of screening tools: reviewing the challenges and opportunities of cost-effectiveness analysis. Public Health Rev **39**, 17 (2018). 10.1186/s40985-018-0093-8 30009081 10.1186/s40985-018-0093-8 PMC6043991

[46] Nakashima, S., et al.: Therapeutic time window of disease-modifying therapy for early Alzheimer’s disease, (in eng). Alzheimers Dement* (N Y)*. **11**(2), e70102 (2025). 10.1002/trc2.70102 10.1002/trc2.70102 PMC12122261 40453977

[47] DiBello, J.R., et al.: Patterns of use of symptomatic treatments for Alzheimer’s disease dementia (AD). BMC Neurol **23**(1), 400 (2023). 10.1186/s12883-023-03447-5 37946118 10.1186/s12883-023-03447-5 PMC10634008

[48] Jutten, R.J., et al.: Finding Treatment Effects in Alzheimer Trials in the Face of Disease Progression Heterogeneity. Neurology **96**(22), e2673–e2684 (2021). 10.1212/WNL.0000000000012022 34550903 10.1212/WNL.0000000000012022 PMC8205463

[49] Shinkins, B., Allen, A.J., Karichu, J., Garrison, L.P., Monz, B.U.: Evidence synthesis and linkage for modelling the cost-effectiveness of diagnostic tests: preliminary good practice recommendations. Appl Health Econ Health Policy **22**(2), 131–144 (2024). 10.1007/s40258-023-00855-z 38316713 10.1007/s40258-023-00855-z PMC10864520

[50] Koster, F., Kok, M.R., Lopes Barreto, D., Weel-Koenders, A.E.A.M.: Capturing patient value in an economic evaluation. Arthritis Care Res (Hoboken) **76**(2), 191–199 (2024). 10.1002/acr.25229 37667586 10.1002/acr.25229

