# Supplementary material for: Cost-effectiveness of testing biofluid biomarkers to diagnose Alzheimer’s disease: a systematic review
Source: Eur J Health Econ. 2025 Dec 12;27(5):1103–15. doi: 10.1007/s10198-025-01877-1 (PMC13451286; doi:10.1007/s10198-025-01877-1)
Supplement: Supplementary file 2 [file 10198_2025_1877_MOESM2_ESM.docx]

**Supplementary Table 2. Full Search Strategies**

**PubMed**

|  | |  | N | |  |
| --- | --- | --- | --- | --- | --- |
| #1 | | (Economic-outcome*[tiab] OR Economic-evaluation*[tiab] OR Return-on-investment*[tiab] OR Cost-effective*[tiab] OR Cost-benefit-analys*[tiab] OR "Cost-Benefit Analysis"[Mesh] OR "Costs and Cost Analysis"[tiab] OR "Cost-Effectiveness Analysis"[Mesh] OR Cost-saving*[tiab] OR quality-adjusted-life-years[tiab] OR QALY[tiab] OR "Quality-Adjusted Life Years"[Mesh]) |  | |  |
|  | |  |  | |  |
| #2 | | ("Biomarkers/cerebrospinal fluid"[Mesh] OR biomarker*[tiab] OR biological-marker*[tiab] OR "Biomarkers/blood"[Mesh]) |  | |  |
|  | |  |  | |  |
| #3 | | ("Alzheimer Disease"[Mesh] OR alzheimer*[tiab] OR Acute-Confusional-Senile-Dementia[tiab]) |  | |  |
|  | |  |  | |  |
| #4 | | #1 AND #2 AND #3 | 319 | |  |
|  | |  |  |  |  |
| **Embase** |  | | |  | |
| #1 | ('alzheimer disease'/exp OR alzheimer*:ti,ab OR 'acute confusional senile dementia':ti,ab) | | | N | |
|  |  | | |  | |
| #2 | (biomarker*:ti,ab OR 'biological marker*':ti,ab OR 'biological marker'/exp) | | |  | |
|  |  | | |  | |
| #3 | ('economic evaluation'/exp OR 'economic outcome*':ti,ab OR 'economic evaluation*':ti,ab OR 'return on investment*':ti,ab OR 'cost effective*':ti,ab OR 'cost benefit analys*':ti,ab OR 'costs and cost analysis':ti,ab OR 'cost saving*':ti,ab OR 'quality adjusted life years':ti,ab OR qaly:ti,ab) | | |  | |
|  |  | | |  | |
| #4 | #1 AND #2 AND #3 | | | 599 | |
|  |  |  |  |  |  |
| **Cochrane** |  | | |  | |
|  |  | | | **N** | |
| #1 | MeSH descriptor: [Cost-Benefit Analysis] explode all trees | | | 11596 | |
| #2 | MeSH descriptor: [Cost-Effectiveness Analysis] explode all trees | | | 142 | |
| #3 | MeSH descriptor: [Quality-Adjusted Life Years] explode all trees | | | 2422 | |
| #4 | (Economic-outcome* OR Economic-evaluation* OR Return-on-investment* OR Cost-effective* OR Cost-benefit-analys* OR "Costs and Cost Analysis" OR Cost-saving* OR quality-adjusted-life-years OR QALY):ti,ab | | | 40217 | |
| #5 | #1 OR #2 OR #3 OR #4 | | | 42511 | |
| #6 | MeSH descriptor: [Biomarkers] explode all trees and with qualifier(s): [cerebrospinal fluid - CF] | | | 182 | |
| #7 | MeSH descriptor: [Biomarkers] explode all trees and with qualifier(s): [blood - BL] | | | 16401 | |
| #8 | (biomarker* OR biological-marker*):ti,ab | | | 43613 | |
| #9 | #6 OR #7 OR #8 | | | 56020 | |
| #10 | MeSH descriptor: [Alzheimer Disease] explode all trees | | | 5452 | |
| #11 | (alzheimer* OR acute-confusional-senile-dementia):ti,ab | | | 13925 | |
| #12 | #10 OR #11 | | | 14537 | |
| #13 | #5 AND #9 AND #12 | | | 19 | |
| **Web of Science** |  | | |  | |
|  |  | | | N | |
| #1 | (TS=(Economic-outcome* OR Economic-evaluation* OR Return-on-investment* OR Cost-effective* OR Cost-benefit-analys* OR "Costs and Cost Analysis" OR Cost-saving* OR quality-adjusted-life-years OR QALY)) | | |  | |
|  |  | | |  | |
| #2 | (TS=(alzheimer* OR acute-confusional-senile-dementia)) | | |  | |
|  |  | | |  | |
| #3 | (TS=(biomarker* OR biological-marker*)) | | |  | |
|  |  | | |  | |
| #4 | #1 AND #2 AND #3 | | | 395 | |
