# Supplementary material for: Cost-effectiveness of testing biofluid biomarkers to diagnose Alzheimer’s disease: a systematic review
Source: Eur J Health Econ. 2025 Dec 12;27(5):1103–15. doi: 10.1007/s10198-025-01877-1 (PMC13451286; doi:10.1007/s10198-025-01877-1)
Supplement: Supplementary file 3 [file 10198_2025_1877_MOESM3_ESM.docx]

**Supplementary Table 3. CHEERS Quality and Risk of Bias Assessment Result**

| **CHEERS Criteria** | **Aye et al.** | **Canestaro et al.** | **Contador et al.** | **Handels et al. (2015)** | **Handels et al. (2017)** | **Lee et al.** | **Michaud et al.** | **Noda et al.** | **Nazco et al.** | **Number of studies fulfilled the criteria** |
| --- | --- | --- | --- | --- | --- | --- | --- | --- | --- | --- |
| Title | Yes | Yes | Yes | Yes | Yes | Yes | Yes | Yes | Yes | 9 |
| Abstract | Yes | Yes | Yes | Yes | Yes | Yes | Yes | Yes | Yes | 9 |
| Introduction: Background and Objectives | Yes | Yes | Yes | Yes | Yes | Yes | Yes | Yes | Yes | 9 |
| Health Economic Analysis Plan | No | No | No | No | No | No | No | No | No | 0 |
| Study Population | Yes | Yes | Yes | Yes | Yes | Yes | Yes | Yes | Yes | 9 |
| Setting and Location | Yes | Yes | Yes | Yes | Yes | Yes | Yes | Yes | Yes | 9 |
| Comparators | Yes | Yes | Yes | Yes | Yes | Yes | Yes | Yes | Yes | 9 |
| Perspective | Yes | Yes | Yes | Yes | Yes | Yes | Yes | Yes | Yes | 9 |
| Time Horizon | Yes | Yes | Yes | Yes | Yes | Yes | Yes | Yes | Yes | 9 |
| Discount Rate | Yes | No | No | Yes | No | Yes | No | Yes | No | 4 |
| Selection of Outcomes | Yes | Yes | Yes | Yes | Yes | Yes | Yes | Yes | Yes | 9 |
| Measurement of Outcomes | Yes | Yes | Yes | Yes | Yes | Yes | Yes | Yes | Yes | 9 |
| Valuation of Outcomes | Yes | Yes | Yes | Yes | Yes | Yes | Yes | Yes | Yes | 9 |
| Measurement and Valuation of Resources and Costs | Yes | Yes | Yes | Yes | Yes | Yes | Yes | Yes | Yes | 9 |
| Currency, Price Date, and Conversion | Yes | Yes | Yes | Yes | Yes | Yes | Yes | Yes | Yes | 9 |

**Supplementary Table 3 - Continued**

| **CHEERS Criteria** | **Aye et al.** | **Canestaro et al.** | **Contador et al.** | **Handels et al. (2015)** | **Handels et al. (2017)** | **Lee et al.** | **Michaud et al.** | **Noda et al.** | **Nazco et al.** | **Number of studies fulfilled the criteria** |
| --- | --- | --- | --- | --- | --- | --- | --- | --- | --- | --- |
| Rationale and Description of Model | Yes | Yes | Yes | Yes | Yes | Yes | Yes | Yes | Yes | 9 |
| Analytics and Assumptions | Yes | Yes | Yes | Yes | Yes | Yes | Yes | Yes | Yes | 9 |
| Characterizing Heterogeneity | Yes | No | No | No | Yes | Yes | No | Yes | No | 4 |
| Characterizing Distributional Effects | No | No | No | No | No | yes | No | No | No | 1 |
| Characterizing Uncertainty | Yes | Yes | Yes | Yes | Yes | Yes | Yes | Yes | Yes | 9 |
| Approach to Engagement | No | No | No | No | No | No | No | No | No | 0 |
| Study Parameters | Yes | Yes | Yes | Yes | Yes | Yes | Yes | Yes | Yes | 9 |
| Summary of Main Results | Yes | Yes | Yes | Yes | Yes | Yes | Yes | Yes | Yes | 9 |
| Effect of Uncertainty | Yes | Yes | Yes | Yes | Yes | Yes | Yes | Yes | Yes | 9 |
| Effect of Engagement | No | No | No | No | No | No | No | No | No | 0 |
| Study Findings, Limitations, Generalizability | Yes | Yes | Yes | Yes | Yes | Yes | Yes | Yes | Yes | 9 |
| Source of Funding | Yes | Yes | Yes | Yes | Yes | Yes | Yes | Yes | Yes | 9 |
| Conflicts of Interest | Yes | Yes | Yes | Yes | Yes | Yes | Yes | Yes | Yes | 9 |
